# Importance, performance frequency, and predicted future importance of dietitians’ jobs by practicing dietitians in Korea: a survey study

**DOI:** 10.3352/jeehp.2024.21.1

**Published:** 2024-01-02

**Authors:** Cheongmin Sohn, Sooyoun Kwon, Won Gyoung Kim, Kyung-Eun Lee, Sun-Young Lee, Seungmin Lee

**Affiliations:** 1 Department of Food & Nutrition, College of Agriculture and Food Sciences, Wonkwang University, Iksan, Korea; 2 Department of Food & Nutrition, Shingu College, Seongnam, Korea; 3 Department of Food & Nutrition, College of Natural Sciences, Seoul Women’s University, Seoul, Korea; 4 Department of Food & Nutrition, College of Biotechnology & Natural Resource., Chung-Ang University, Anseong, Korea; 5 Department of Food & Nutrition, Health & Wellness College, Sungshin Women’s University, Seoul, Korea; Hallym University, Korea

**Keywords:** Counseling, Dietetics, Menu planning, Nutritionists, Republic of Korea

## Abstract

**Purpose:**

This study aimed to explore the perceptions held by practicing dietitians of the importance of their tasks performed in current work environments, the frequency at which those tasks are performed, and predictions about the importance of those tasks in future work environments.

**Methods:**

This was a cross-sectional survey study. An online survey was administered to 350 practicing dietitians. They were asked to assess the importance, performance frequency, and predicted changes in the importance of 27 tasks using a 5-point scale. Descriptive statistics were calculated, and the means of the variables were compared across categorized work environments using analysis of variance.

**Results:**

The importance scores of all surveyed tasks were higher than 3.0, except for the marketing management task. Self-development, nutrition education/counseling, menu planning, food safety management, and documentation/data management were all rated higher than 4.0. The highest performance frequency score was related to documentation/data management. The importance scores of all duties, except for professional development, differed significantly by workplace. As for predictions about the future importance of the tasks surveyed, dietitians responded that the importance of all 27 tasks would either remain at current levels or increase in the future.

**Conclusion:**

Twenty-seven tasks were confirmed to represent dietitians’ job functions in various workplaces. These tasks can be used to improve the test specifications of the Korean Dietitian Licensing Examination and the curriculum of dietetic education programs.

## Graphical abstract

[Fig f1-jeehp-21-01]

## Introduction

The Korean Dietetic Association (KDA) defines a dietitian as “a food and nutrition expert with a legal qualification who performs food service management and provides nutrition services for individuals, organizations, and communities to prevent diseases and promote health” [1]. To be qualified as a dietitian, one must complete a degree program specified by the Ordinance of the Ministry of Health and Welfare and must pass the Korean Dietitian Licensing Examination (KDLE) [2].

Most Korean dietitians are employed in hospitals, schools, businesses/industries, public health centers, social welfare facilities, and centers for children’s food service/social welfare food service [1]. Environmental changes including the aging population, changes in lifestyles and dietary habits, an increase in public interest in health and nutrition, and sustainable food production and food security require dietitians to perform more diverse roles than before and to change how they provide services [3-5]. It is periodically necessary to review the kinds of work performed by dietitians, since the test items of the KDLE should be developed based on dietitians’ current tasks in order to identify potential dietitians who are able to meet the requirements of a contemporary workplace.

The purpose of this study was to explore the current job functions of Korean dietitians prior to revising specifications for job-based test item development for the KDLE. We examined dietitians’ perceived importance of tasks in their own work environments, the frequencies at which those tasks are carried out, and predictions regarding the importance of those tasks in future work environments.

## Methods

### Ethics statement

This research was approved by the Institutional Review Board of Wonkwang University (WKIRB-202306-SB-044). Informed consent was obtained from participants.

### Study design

This was a cross-sectional survey study.

### Setting

A total of 1,000 KDA members who were selected randomly in dietetic practice groups representing various work environments received an invitation email that contained an online survey link (www.moaform.com) and participated in the survey voluntarily from June 30 to July 6, 2023.

### Participants

The study population was made up of practicing dietitians who are also members of the KDA. A total of 350 dietitians voluntarily participated in the survey.

### Variables

Dietitians’ perceived importance, performance frequency, and predicted future importance of 27 tasks were outcome variables to be measured.

### Data sources/measurement

For the survey questionnaires, the authors reviewed the literature on the dietitians’ job analysis [3,6-8] and identified 5 duties (nutrition management, food service management/food safety management, community nutrition management, organizational management, and professional development) and 27 tasks pertaining to those duties for the questionnaire. For each task, the dietitians were asked to rate perceived importance and performance frequencies in their own position. To understand the changing work environment, they were asked to predict how the importance of the tasks would change in the future.

The perceived importance of the tasks was rated using a 5-point Likert-type scale (1: very unimportant to 5: very important). The performance frequency was rated based on a 5-point Likert-type scale (1: do not perform at all to 5: perform very frequently). The participants were also asked to predict how the importance of the tasks would change in the future using a 5-point Likert-type scale (1: will become very unimportant in the future to 5: will become very important in the future) (Supplement 1).

### Bias

KDA members participated in the online survey voluntarily. The survey was closed when it reached the target number of respondents. Therefore, there may have been some selection bias.

### Study size

Since all voluntary participants were targeted, no sample size estimation was done.

### Statistical methods

The collected data were analyzed using IBM SPSS ver. 25.0 (IBM Corp.). Descriptive statistics were calculated, and the means of the variables were compared by work setting using one-way analysis of variance. When a significant difference was found, the Duncan post-hoc test was performed with a significance level of 0.05. The raw data are available from Dataset 1.

## Results

### Participants

Most of the respondents were female (98%), and 35% were aged between 31 and 40 years (Table 1). The average length of employment was 13.6 years. Sixty-eight percent of respondents were employed in schools/kindergartens/daycare centers (25.4%), hospitals (22.6%), and public health centers (20.0%).

### Main results

The tasks with high importance scores were self-development (4.22), nutrition education/counseling (4.16), menu planning (4.09), food safety management (4.09), and documentation/data management (4.07) (Table 2). With the exception of marketing management (2.99), all tasks were rated higher than 3.0. The performance frequency scores ranged from 2.33 to 4.24; the highest score was related to documentation/data management and the lowest score was related to community nutrition program planning. When asked to predict how the importance of the tasks would change in the future, all listed tasks were rated 3.50 or higher (with a score of 3 meaning that the respondent believes the current level of importance of the task will remain unchanged in the future). The dietitians perceived that the importance of tasks related to nutrition management, such as nutrition assessment/diagnosis, nutrition management planning, nutrition education/counseling, and nutrition support, would increase in the future. In addition, food and nutrition research was also expected to become more important.

The importance of 27 tasks was compared collectively under representative duties across the workplaces. The importance scores of all duties, with the exception of professional development, differed significantly by work setting (Table 3). The nutrition management duty was rated significantly higher by dietitians working in hospitals and public health centers (P<0.001); while the duty of food service management was rated significantly higher by those working in schools/kindergartens/daycare centers, business/industries/contract management companies, and social welfare institutions (P<0.001). The duty of community food and nutrition service was rated significantly higher by those working in public health centers (P<0.001). The duty of organizational management was rated significantly lower by hospital dietitians (P=0.001).

Within any given work environment, there was a similar tendency between the high perception of importance of a duty and the high frequency at which that duty was performed. Dietitians at hospitals and public health centers performed the duty of nutrition management with a high degree of frequency and placed a high level of importance on the duty. Likewise, dietitians working in schools/kindergartens/daycare centers, business/industries/contract management companies, and social welfare institutions performed tasks related to the duty of food service management/safety management with a high degree of frequency and placed a high level of importance on the collective tasks falling under that duty (P<0.001). Perceptions of future changes in the importance of the duties differed significantly by the work environment and often did not correspond to the current perceptions of importance and frequency of performance, but the mean scores were all 3.0 points or higher regardless of the work environment.

## Discussion

### Key results/interpretation

The purpose of this study was to explore the current job functions of dietitians and to identify future needs in their work environments. In total, 350 practicing dietitians participated in the survey. The tasks of the study were considered to represent dietitians’ jobs, since the importance scores of all except one were rated higher than 3.0 on a 5-point Likert scale.

The overall importance of collective tasks falling under specific duties differed by work environment, with the exception of those tasks comprising the duty of professional development. The collective scores pertaining to a high frequency of performance of tasks comprising a given duty corresponded to the collective scoring of the perceived importance of said duty across work environments. This means that dietitians perceived the tasks performed more frequently as more important. Dietitians in schools/kindergartens/daycare centers evaluated the importance of food service management/safety management and organizational management highly; correspondingly, they performed the collective tasks of both these duties frequently. The importance of organizational management was rated lower by dietitians working in hospitals than by dietitians in other workplaces. While many dietitians work as the sole dietitian in schools/kindergartens/daycare centers, social welfare institutions, and business/industries food service operations, multiple dietitians can be found in a hospital, with one designated dietitian overseeing management functions.

In terms of the future importance of the tasks surveyed, the respondents perceived that all tasks would become more important or at least remain at their current levels of importance in the dietitians’ future work environments. In order to select dietitians with the capabilities to meet the changing demands for dietetics, the test specification of KDLE should be based on the jobs that dietitians perform. Based on the findings of our study, current test items and item development specifications need to be reviewed.

Documentation/data management was the only task that was evaluated higher than 4.0 for both importance and performance frequency. This means that this task is both important and performed frequently by dietitians in all work environments. In addition, it is expected to be important in future work environments. However, competencies related to this task cannot be appropriately evaluated on a pencil-paper test. Instead, related knowledge and skills can be integrated into various courses and projects of undergraduate programs. The collective tasks comprising the duty of professional development were evaluated as important across all the work environments. In an optional open-ended question, many respondents answered that competencies related to identifying new scientific findings and trends and incorporating them into dietetic practice were important. The related competencies should be obtained through continuing education during a dietitian’s career as well as through undergraduate programs.

### Comparison with previous studies

The tasks with high importance scores in the study, such as menu planning, food safety management, and nutrition education/counseling, were reported as important in previous research [3,7,8]. In a study with dietitians working in schools, determining nutritional standards, inspecting products, preparing production schedules and work management, planning menus and preparing purchase orders, educating food service staff on safety education, and teaching in class were rated as highly important tasks [8]. Similar to the findings of this study, Choi and Lee [3] reported that the task of marketing management received the lowest importance score among Korean dietitians. Documentation/data management was the most frequently performed task in this study; the performance frequencies of office work administration, performing paperwork, and preparing reports, which were related to documentation/data management, were rated highly by dietitians [3,8].

A systematic review by Blair et al. [4] reported 52 emerging or expanding areas of dietetic practice, including aged care, agriculture, animal nutrition, author, business, catering, and corporate wellness. To meet current and future needs, they also suggested 21 different skills, including clinical skills, social media skills, business skills, collaboration, client/customer focus, computer literacy, and financial management. In this study, the tasks of self-development and food and nutrition research were rated important and expected to be more important in the future. Dietitians seemed to be well aware of the need to prepare for a changing work environment.

### Limitations

The respondents were recruited through the KDA and participated in the survey voluntarily. The survey was closed when it reached the target number of respondents. Therefore, the age, work experience, and workplace distribution of respondents may not represent the actual employment distribution of practicing dietitians. However, respondents were recruited from a national sample with more than 30 workplace environments represented. The findings may not be generalizable, but they do reflect the various natures of dietitians’ job functions.

### Implications

To improve the KDLE for selecting competent dietitians, the test specifications should be revised and the questions of the test item inventory can be reviewed based on the findings of the study. In addition to the content of the test items, the number of items per test subject should be revised. To facilitate the survey, the study questions were limited to tasks. However, there is a need for agreement on the task elements comprising each task so that test writers can develop valid task-based items. Lastly, both dietetics educators and practitioners need to be involved in the test specification review and item development process.

### Conclusion

The tasks that were analyzed in the study were evaluated as important and performed frequently in current work environments and would either continue to be or become more important in the future. Therefore, these tasks can be used for updating testing materials covered by the KDLE. Dietetic professionals work in rapidly changing and evolving environments. The test specifications of the KDLE need to be revised and updated periodically to reflect the professional requirements of dietitians. Not all competencies related to the tasks are testable by the KDLE, but they are still important. In such cases, it is desirable to receive education through an undergraduate curriculum or continuing education after obtaining a license.

## Figures and Tables

**Figure f1-jeehp-21-01:**
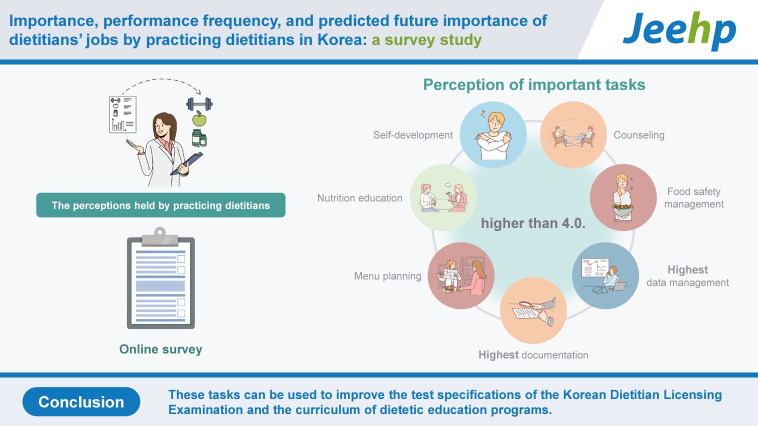


**Table 1.** General characteristics of the respondents

Characteristic	No. (%)
Age (yr)	
≤30	66 (18.9)
31–40	122 (35.0)
41–50	91 (26.1)
≥51	70 (20.1)
Work experience (yr)	
≤5	84 (24.1)
6–10	85 (24.4)
11–15	60 (17.2)
16–20	31 (8.9)
21–25	32 (9.2)
≥26	67 (16.3)
Work setting	
Schools/kindergartens/daycare centers	89 (25.4)
Hospitals	79 (22.6)
Public health centers	70 (20.0)
Centers for children’ food service/social welfare food service	48 (13.7)
Business & industry food service/contract food service companies	32 (9.1)
Social welfare centers	32 (9.1)

**Table 2.** Perceived importance, performance frequency, and expected change of importance scores of tasks among Korean dietitians

Duty	Task	Current importance^a)^	Frequency of performance^b)^	Change of importance^c)^
Nutrition management	Nutrition assessment and diagnosis	3.63±1.30	3.16±1.43	4.16±0.93
Nutrition management	Nutrition management planning	3.92±1.11	3.26±1.32	4.09±0.94
Nutrition management	Nutrition education and counseling	4.16±1.03	3.67±1.21	4.23±0.93
Nutrition management	Nutrition support	3.50±1.28	2.78±1.37	4.03±1.00
Nutrition management	Nutrition monitoring	3.50±1.23	3.10±1.46	3.92±1.02
Nutrition management	Nutrition evaluation	3.61±1.20	3.15±1.41	3.99±0.98
Food service management/safety management	Menu planning	4.09±1.19	3.57±1.43	3.95±1.00
	Purchasing	3.67±1.32	2.97±1.52	3.54±1.03
	Food ingredient management (receiving and storage management)	3.83±1.34	3.38±1.64	3.65±1.08
	Food production/service management	3.72±1.43	3.44±1.70	3.63±1.06
	Equipment and facility management	3.55±1.43	3.20±1.62	3.52±1.05
	Foodservice operations planning and evaluation	3.70±1.34	2.87±1.41	3.74±1.04
	Food safety management	4.09±1.31	3.63±1.51	3.93±1.03
	Workplace safety management	3.76±1.34	3.12±1.48	3.84±1.05
Community nutrition management	Community need assessment	3.39±1.37	2.43±1.37	3.78±0.99
	Community nutrition program planning	3.28±1.39	2.33±1.33	3.74±1.02
	Nutrition service in community	3.27±1.40	2.52±1.48	3.78±1.01
	Networking in community	3.29±1.33	2.42±1.30	3.77±1.00
	Program evaluation	3.25±1.41	2.39±1.38	3.70±1.01
Organizational management	Finance/accounting management	3.86±1.24	3.44±1.30	3.61±0.98
	Human resource management	3.84±1.25	3.26±1.36	3.77±0.97
	Marketing management	2.99±1.32	2.51±1.34	3.61±0.99
	Documentation/data management	4.07±1.03	4.24±1.03	3.60±0.96
Professional development	Cooperation with various stakeholders	3.57±1.21	2.82±1.21	3.61±0.93
	Work improvement activities	3.89±1.01	3.21±1.10	3.80±0.92
	Food and nutrition research	3.86±1.03	3.29±1.17	4.13±0.91
	Self-development	4.22±0.88	3.29±0.99	3.92±0.96

Values are presented as mean±standard deviation.

^a)^A 5-point Likert-type scale was used from 1 (very unimportant) to 5 (very important). ^b)^A 5-point Likert-type scale was used from 1 (do not perform at all) to 5 (perform very frequently). ^c)^A 5-point Likert-type scale was used from 1 (will become very unimportant in the future) to 5 (will become very important in the future).

**Table 3.** Comparisons of perceived importance, performance frequency, and change of importance of tasks among Korean dietitians by work environment

Duty	Schools/kindergartens/daycare centers (n=89)	Hospitals (n=79)	Public health centers (n=70)	Business and industry food service/contract management companies (n=32)	Social welfare institutions (n=32)	Centers for children’s food service/social welfare food service (n=48)	P-value
Current importance^a)^							
Nutrition management	3.48±0.85^b^	4.18±0.99^c^	4.26±0.79^c^	2.76±1.07^a^	3.43±1.04^b^	3.48±0.91^b^	<0.001
Food service management/safety management	4.50±0.55^c^	3.59±1.15^b^	2.75±1.21^a^	4.39±0.96^c^	4.48±0.55^c^	3.51±0.82^b^	<0.001
Community nutrition management	2.87±1.23^a^	2.65±1.32^a^	4.43±0.93^d^	2.69±1.12^a^	3.36±1.07^b^	3.86±0.95^c^	<0.001
Organizational management	3.65±0.80^b^	3.29±0.94^a^	3.77±1.09^b^	3.90±0.79^b^	3.70±0.69^b^	3.98±0.85^b^	0.001
Professional development	4.06±0.73	3.84±0.79	4.14±0.89	4.00±0.76	3.92±0.76	3.92±0.88	0.257
Frequency of performance^b)^							
Nutrition management	2.81±0.95^bc^	3.85±1.22^d^	3.87±0.86^d^	2.22±1.09^a^	3.01±1.00^c^	2.56±1.00^ab^	<0.001
Foodservice management/safety management	4.22±0.62^c^	2.97±1.26^b^	1.95±1.04^a^	4.27±1.02^c^	4.18±0.60^c^	2.68±0.87^b^	<0.001
Community nutrition management	1.86±1.09^a^	1.71±1.00^a^	3.67±1.07^d^	2.31±1.12^b^	2.51±1.04^bc^	2.81±1.07^c^	<0.001
Organizational management	3.31±0.77^b^	2.82±0.95^a^	3.31±1.09^b^	3.81±0.72^c^	3.31±0.70^b^	3.35±0.80^b^	<0.001
Professional development	3.28±0.82^a^	3.19±0.81^a^	3.30±1.01^a^	3.72±0.90^b^	3.27±0.62^a^	3.01±0.89^a^	0.018
Change of importance^c)^							
Nutrition management	3.79±0.81^a^^b^	4.36±0.74^c^	4.36±0.76^c^	3.50±0.91^a^	4.09±0.80^bc^	4.07±0.95^bc^	<0.001
Food service management/safety management	3.84±0.81^a^^b^	3.53±0.67^a^	3.53±1.25^a^	4.14±0.78^b^	4.08±0.76^b^	3.62±0.74^a^	0.001
Community nutrition management	3.44±0.95^a^^b^	3.63±0.83^a^^b^	4.29±0.91^d^	3.33±1.02^a^	3.74±0.87^bc^	4.06±0.80^cd^	<0.001
Organizational management	3.52±0.77^a^^b^	3.34±0.64^a^	3.95±0.95^c^	3.80±0.73^bc^	3.76±0.69^bc^	3.73±0.76^bc^	<0.001
Professional development	3.84±0.82^a^	3.88±0.74^a^	4.22±0.88^b^	3.90±0.79^a^^b^	3.87±0.75^a^	3.98±0.76^b^	0.048

A different superscript in the same row means a significant difference at the significance level of 0.05 by the Duncan post-hoc test.

^a)^A 5-point Likert-type scale was used from 1 (very unimportant) to 5 (very important). ^b)^A 5-point Likert-type scale was used from 1 (do not perform at all) to 5 (perform very frequently). ^c)^A 5-point Likert-type scale was used from 1 (will become very unimportant in the future) to 5 (will become very important in the future).

## Footnotes

**Data availability**
Data files are available from Harvard Dataverse: https://doi.org/10.7910/DVN/3NVNBU Dataset 1. Raw data of responses from 350 participants.

## Supplementary materials

Supplementary files are available from Harvard Dataverse: https://doi.org/10.7910/DVN/3NVNBU

Supplement 1. A survey questionnaire on the importance, performance frequency, and predicted future importance of dietitians’ jobs in Korea.

Supplement 2. Audio recording of the abstract.

## References

[1] The Korean Dietetic Association (2023). Definition of dietitian [Internet]. https://www.dietitian.or.kr/work/introduction/ki_about.do.

[2] National Nutrition Management Act (2020). Law No. 17472 (Aug 11, 2020) [Internet]. https://www.law.go.kr/법령/국민영양관리법.

[3] Choi JH, Lee KE (2019). Job analysis of dietitian using DACUM technique-importance, performance, and difficulty analysis. Korean J Food Nutr.

[4] Blair M, Mitchell L, Palermo C, Gibson S (2022). Trends, challenges, opportunities, and future needs of the dietetic workforce: a systematic scoping review. Nutr Rev.

[5] Academy Quality Management Committee (2018). Academy of Nutrition and Dietetics: revised 2017 scope of practice for the registered dietitian nutritionist. J Acad Nutr Diet.

[6] Seo WK, Rho JO (2020). Study on the job importance, job performance, job satisfaction, and turnover intention of employees according to the budget size of Center for Children’s Foodservice Management (CCFSM). J Korean Soc Food Sci Nutr.

[7] Cha JA, Kim KE, Kim EM, Park MS, Park YK, Baek HJ, Lee SM, Choi SK, Seo JS (2013). Development of job description of clinical dietitians in hospitals by the DACUM method. J Korean Diet Assoc.

[8] Kim SH, Lee KE, Kim JS (2016). Job perception and the need for job improvement among school nutrition teachers in Seoul. Korean J Community Nutr.

