# Supplementary figures and images for: Importance, performance frequency, and predicted future importance of dietitians’ jobs by practicing dietitians in Korea: a survey study
Source: J Educ Eval Health Prof. 2024 Jan 2;21:1. doi: 10.3352/jeehp.2024.21.1 (PMC11894033; doi:10.3352/jeehp.2024.21.1)

**
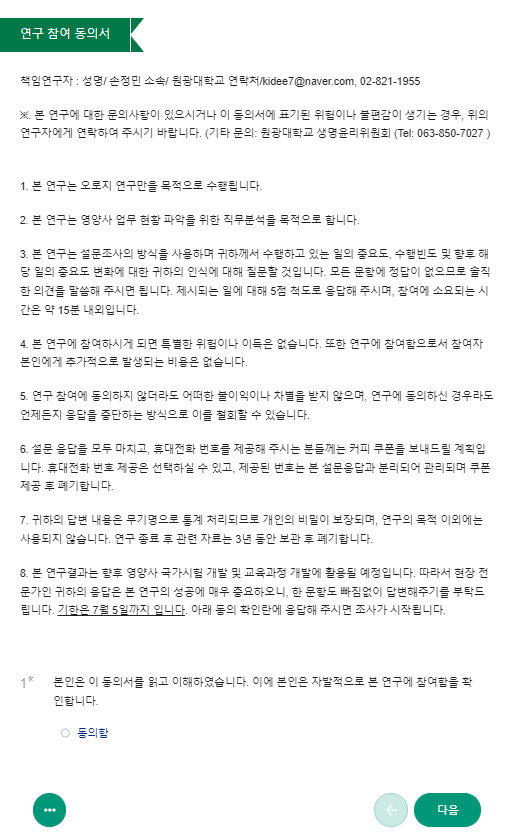
**

**
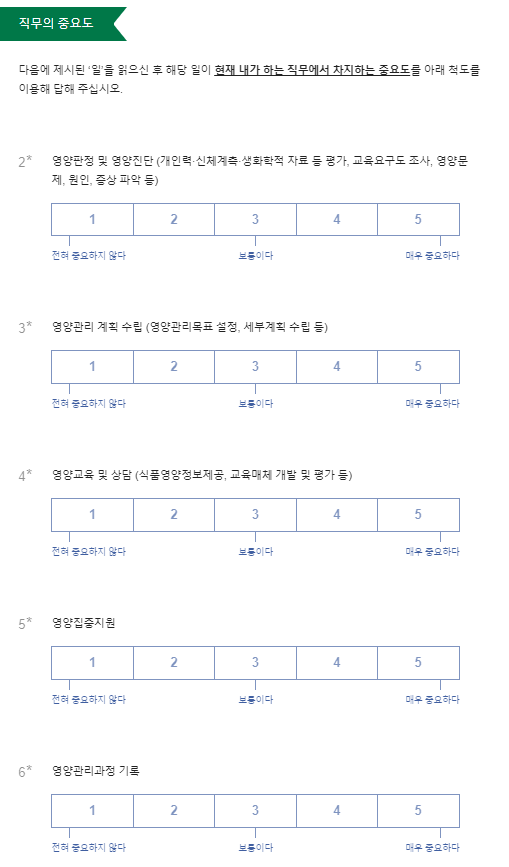
**

**
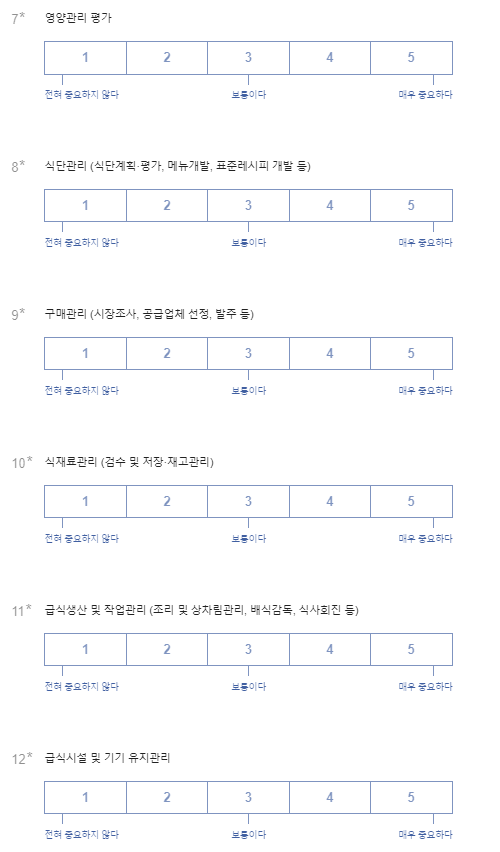
**

**
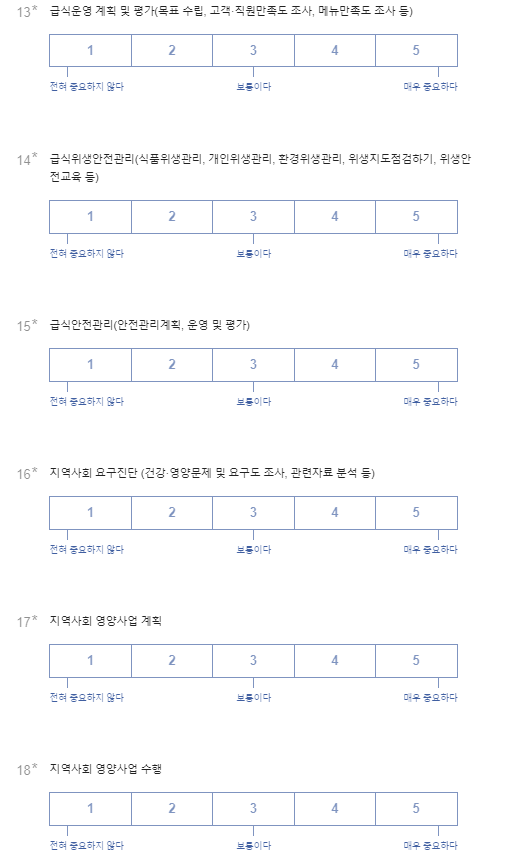
**

**
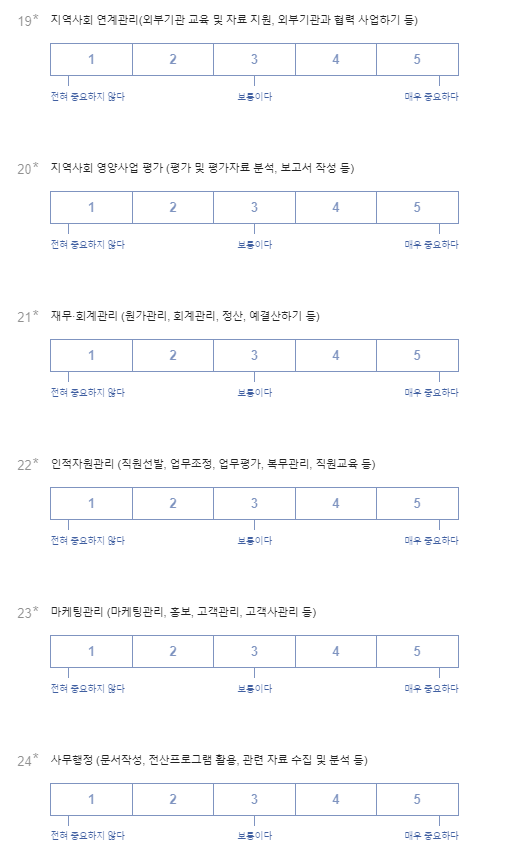
**

**
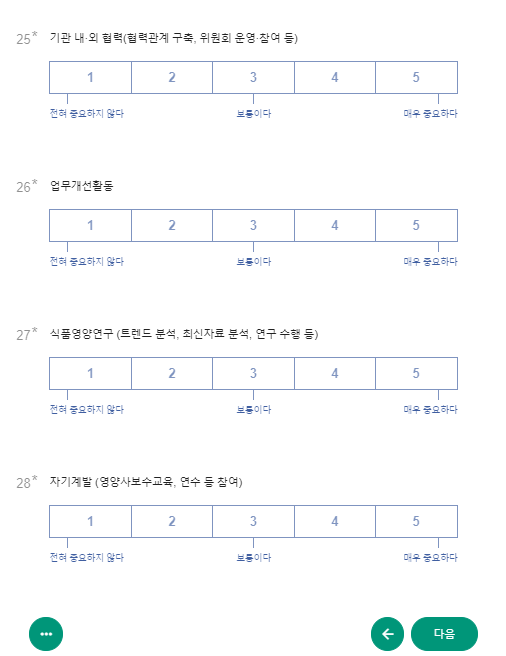
**

**
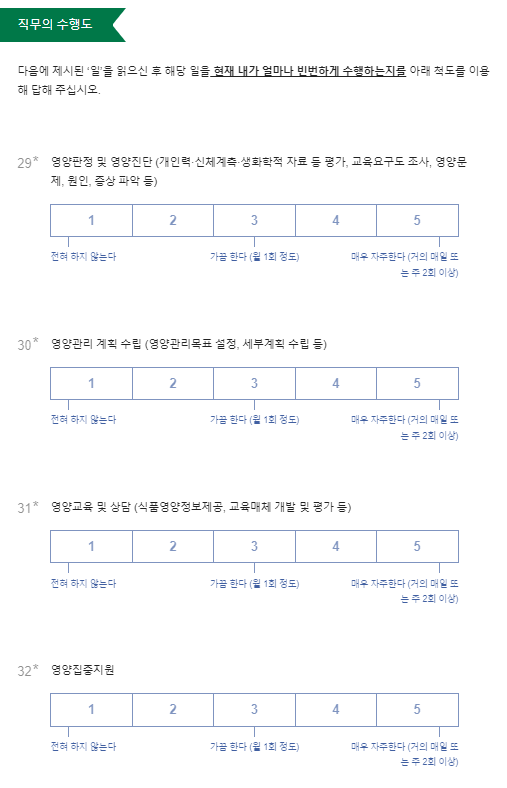
**

**
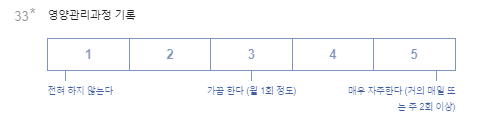
**

**
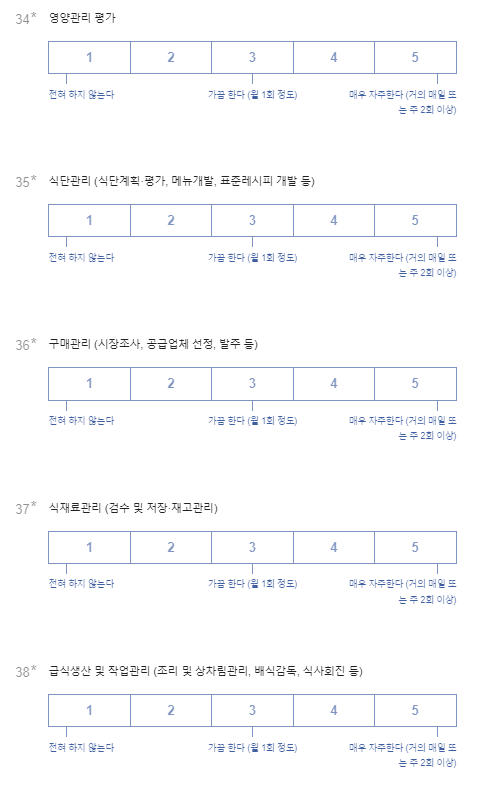

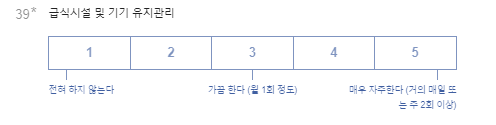
**

**
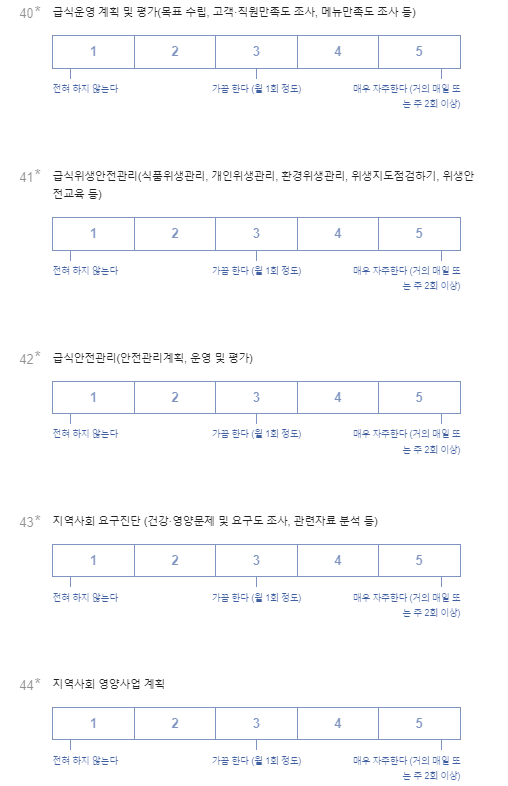
** **
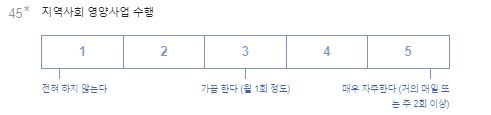
**


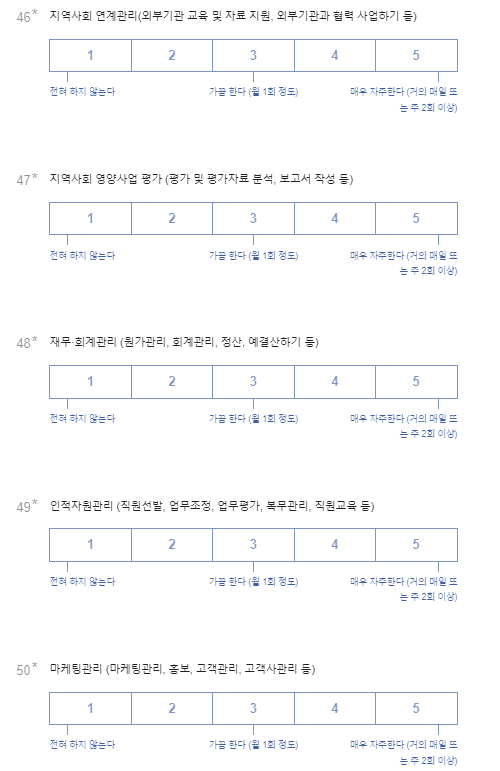


**
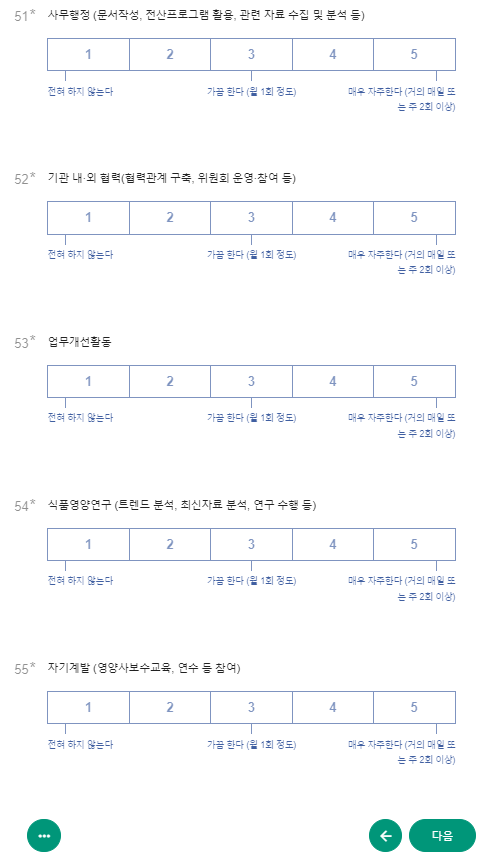
**

**
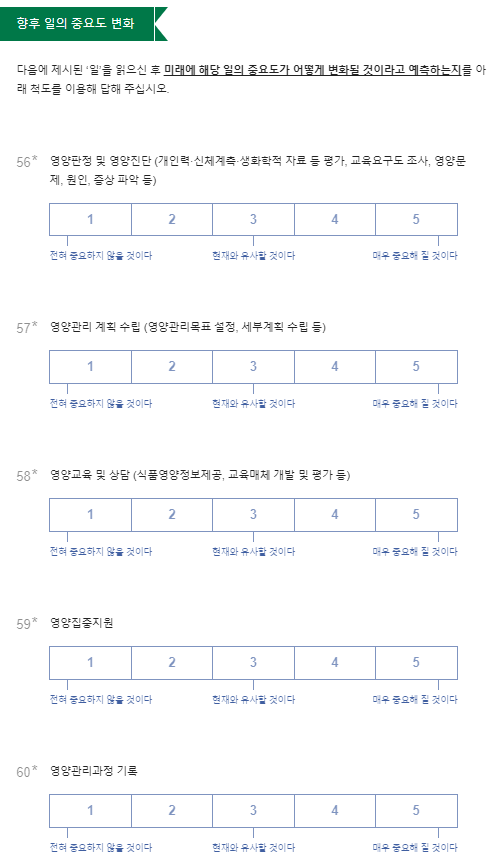
**

**
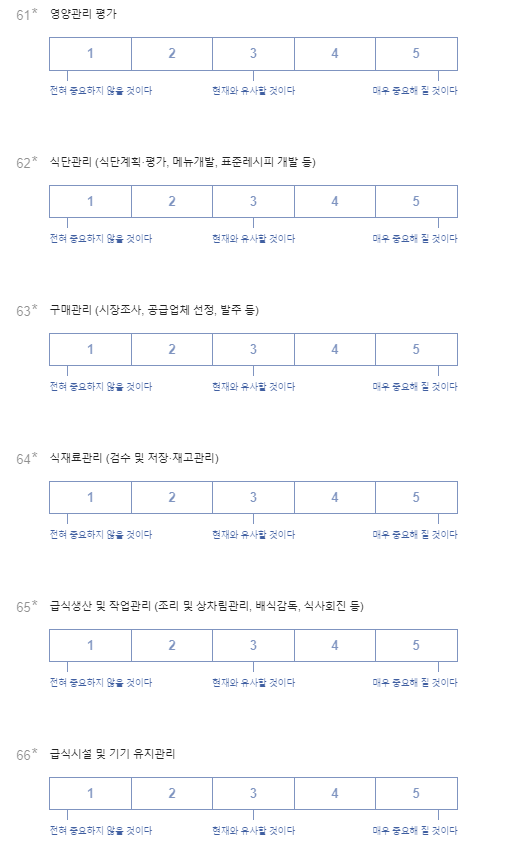
**

**
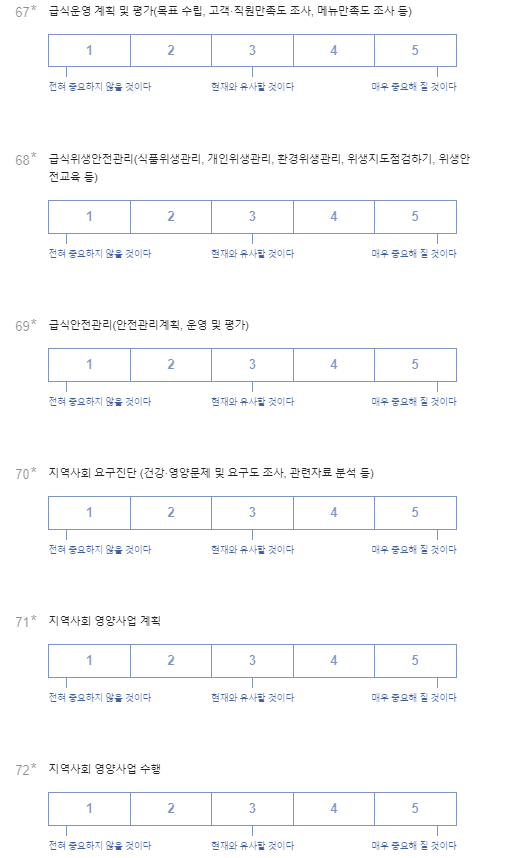
**

**
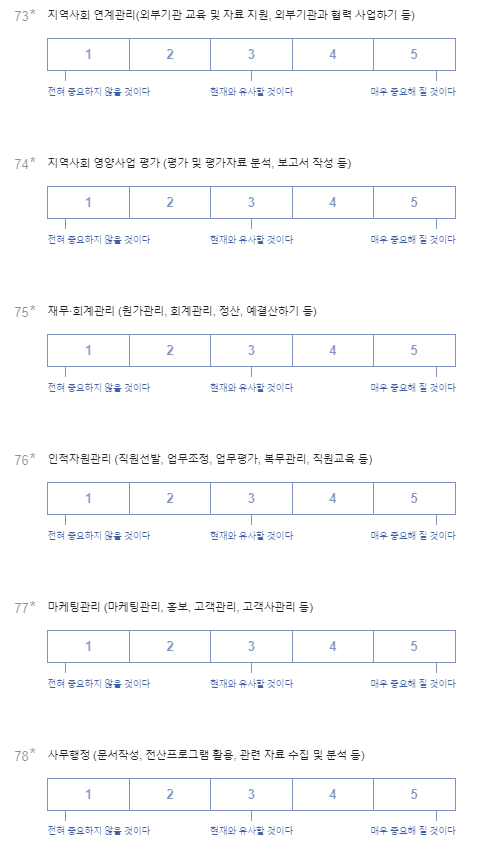
**

**
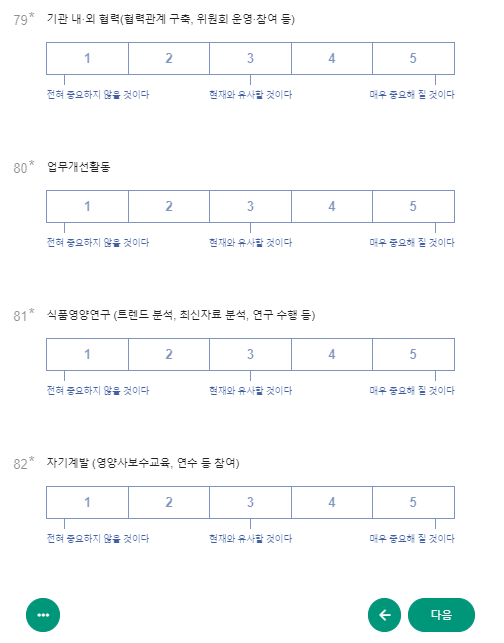
**

**
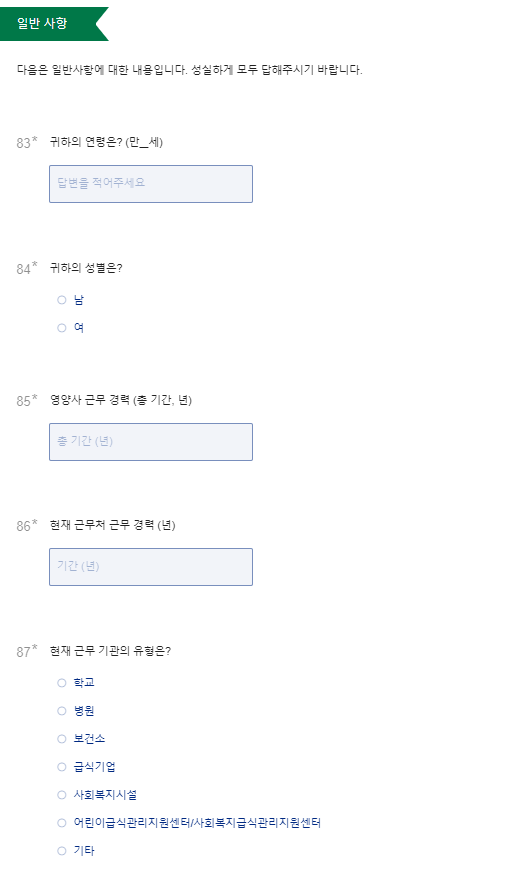
**

**
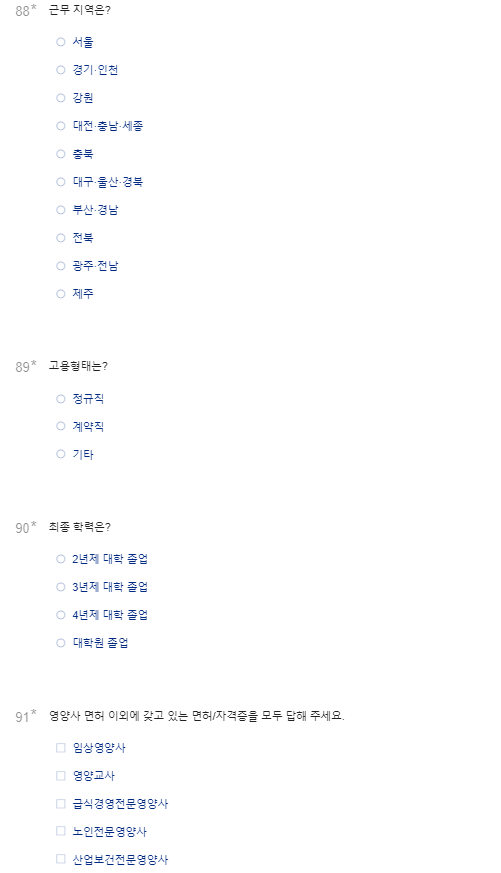
** **
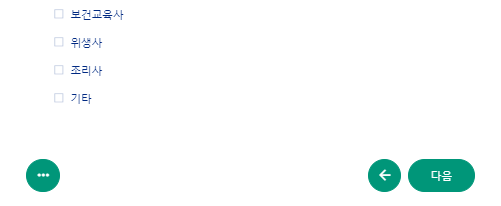
**

**
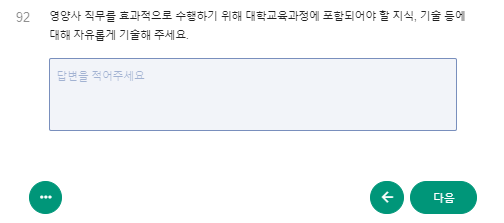
**

**
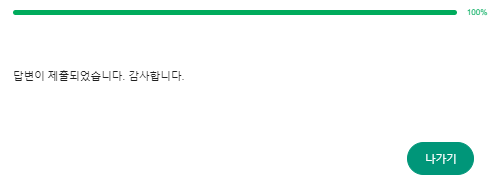
**

Supplement: Supplementary file 2 — Supplement 1. A survey questionnaire on the importance, performance frequency, and predicted future importance of dietitians’ jobs in Korea. [file jeehp-21-01-suppl1.docx]
